# Circulating Heat Shock Protein 70 in Health, Aging and Disease

**DOI:** 10.1186/1471-2172-12-24

**Published:** 2011-03-28

**Authors:** Rose Njemini, Ivan Bautmans, Oscar O Onyema, Katrien Van Puyvelde, Christian Demanet, Tony Mets

**Affiliations:** 1 Gerontology & Frailty in Aging (FRIA) research group, Faculty of Medicine and Pharmacy, Vrije Universiteit Brussel, Brussels, Belgium; 2 Geriatric Unit, Universitair Ziekenhuis Brussel, Brussels, Belgium; 3 Immunology laboratory, Universitair Ziekenhuis Brussel, Brussels, Belgium

## Abstract

**Background:**

Heat shock proteins (Hsp) are ubiquitously synthesised in virtually all species and it is hypothesised that they might have beneficial health effects. Recent studies have identified circulating Hsp as an important mediator in inflammation - the effects of low-grade inflammation in the aging process are overwhelming. While much is known about intracellular Hsp70, scant data exist on circulating Hsp70 in the aging context. Therefore, the objectives of this study were to investigate the effect of age and disease on circulating Hsp70 and, in particular, to evaluate the association between circulating Hsp70 and inflammatory parameters.

**Results:**

Serum Hsp70, Interleukin (IL) -10, IL-6 and Tumor Necrosis Factor (TNF) alpha concentrations were determined in 90 hospitalised geriatric patients (aged 83 ± 6 years) and in 200 community-dwelling control subjects (100 elderly, aged 74 ± 5 years, and 100 young, aged 23 ± 3 years). In the community-dwelling elderly, serum Hsp70 and IL-10 concentrations were significantly lower and IL-6 was significantly higher when compared to healthy young control subjects. Elderly patients presenting inflammation (CRP serum levels ≥5 mg/L) showed significantly (p = 0.007) higher Hsp70 values; and Hsp70 correlated positively (p < 0.001) with IL-6 and CRP, but not with TNF-alpha or IL-10. A significant association was also noted between Hsp70 levels and the degree of dependency and cognitive decline in geriatric patients.

**Conclusions:**

The present data provide new evidence that serum concentration of Hsp70 decreases with age in a normal population. Our study also shows that higher levels of Hsp70 are associated with inflammation and frailty in elderly patients.

## Background

Heat shock proteins (Hsp) are primarily expressed as intracellular proteins that exhibit a range of fundamental roles necessary for cell survival. Intracellular functions of Hsp include both specific biological actions that are linked to a particular Hsp and more general chaperone activities that result in the stabilisation and protection of newly synthesised proteins, targeting ultimately damaged proteins for degradation and transportation of proteins to their sites of activity [1]. Although research has mainly focused on intracellular Hsp, it is now obvious that Hsp [2] as well as Hsp receptors [3] are expressed on the cell surface and that Hsp are circulating in the blood [4]. Hsp can be secreted by viable cells, such as peripheral blood mononuclear cells which are thought to contribute significantly to circulating Hsp70 [5,6]. Extracellular Hsp have been reported to exhibit beneficial properties, such as enhancing the survival of cultured cells [7], and mitigating autoimmune disorders [8-10]. The prevention or arrest of inflammatory damage by Hsp is thought to involve T cell reactivity, resulting in the enhancement and/or mitigation of specific cytokine profiles [8,11,12].

From a biological point of view, aging is associated with a decrease in the homeostatic mechanisms that promote adaptive responses to challenges. Numerous studies have attested to the increased vulnerability of the elderly to inflammatory challenges and their diminished capacity to respond to stress. Studies *in vitro *and in animal models have demonstrated that the stress response and the capacity to produce Hsp decrease with aging [4,13-15]. However, we have previously drawn attention to the higher basal levels of Hsp70 that are found in unstimulated lymphocytes and monocytes of the elderly compared to young healthy volunteers [16]. This observation might indicate that the Hsp system is in a more activated state in elderly people. Despite the well-described age-related changes at the cellular level, scant information is available on the Hsp present in the circulation [4,17,18]. We reported previously that inflammation in elderly patients resulted in higher circulating Hsp70 levels [17,19,20]. However, it remains unclear whether the concentrations of circulating Hsp change as a person gets older, and whether there is any relationship with the subclinical inflammatory condition that accompanies normal aging. Here we report on the serum levels of Hsp70 and the relationship with pro- and anti-inflammatory cytokines in a large number of normal young and elderly volunteers. Since aging results in an increased prevalence of various diseases that might interfere with Hsp production, we also investigated a group of geriatric patients.

## Methods

### Participants

We included 90 elderly patients (60 women and 30 men, aged between 60 and 93 years, mean age 83.4 years (SD 5.6)), hospitalised consecutively in the geriatric unit at the Universitair Ziekenhuis Brussel. For the evaluation of the basic activities of daily living (bADL) a modified scale was used, according to Katz et al. [21], to assess the six bADL items (bathing, dressing, toileting, ambulating or transferring, continence and feeding), on a four-point scale ranging from completely independent (1) to completely dependent (4). The Mini Mental State Evaluation (MMSE) [22] was used to evaluate the cognitive status of these patients, whenever the patient could cooperate adequately. This tool, using a scoring system between 0 and 30, covers five cognitive domains (orientation, memory, attention, language and praxis) enabling the rapid evaluation of cognitive functioning, with a fairly high sensitivity, specificity and reproducibility.

Control groups comprised 100 physically independent community-dwelling older subjects (51 women and 49 men, aged between 61 and 86 years, mean age 74.4 years (SD 4.6)), recruited from our research department's database of elderly volunteers and from senior citizens' organisations, and 100 young subjects (51 women and 49 men, aged between 20 and 30 years, mean age 23 years (SD 2.9)), recruited from among the staff and students of the Vrije Universiteit Brussel. We excluded volunteers who had a chronic inflammatory pathology or acute or uncontrolled conditions but, as recommended by current guidelines [23], comorbidity was not an exclusion criterion per se. Participants who received anti-inflammatory medication other than low-dose aspirin, used as a platelet antiaggregant, were excluded.

The study was approved by the Vrije Universiteit Brussel's local ethical committee and all participants gave their written informed consent.

### Blood sampling

Hospitalised patients were assessed for participation in the study within the first five days after admission. If subjects participated in sports, measurements were performed at least 12 hours after their last intensive physical activity. Venous blood samples were taken in the morning from all participants; sera were separated from blood cells and stored at -20°C.

### Sandwich ELISA for Hsp70 determination in serum

Hsp70 in serum was detected using a commercial ELISA kit (StressGen, Canada), as previously described, with minor modifications [24]. All reagents, dilutions and calculations were applied according to the manufacturer's instructions. The standard curve ranged from 0.20 ng/mL to 12.5 ng/mL with a sensitivity of 90 pg/mL.

### Other determinations

Sera were assayed for IL-6, TNF-alpha, IL-10 and CRP using commercial ELISA kits (Invitrogen, Merelbeke, Belgium). Intra-assay precision expressed as a coefficient of variance (CV), as determined by the manufacturer for low (L), normal (N) and high (H) standards, were: for IL-6: CV-L = 7.7%, CV-N = 5.7%, CV-H = 5.1%; for TNF-α: CV-L = 5.2%, CV-N = 4.1%, CV-H = 3.9%, and for IL-10: CV-L = 2.9%, CV-N = 2.9%, CV-H = 4.8%. For CRP, the CV-L was 1.3% and CV-H 2.1%. Serum samples with a concentration of IL-6 lower than 7.8 pg/mL, which was the lower detection limit for the standard kit, were re-analysed using the high-sensitivity IL-6 kit (Invitrogen, Merelbeke, Belgium) with a detection limit of 0.16 pg/mL, and CV-L = 8.3%, CV-N = 6.2%, CV-H = 4.7%. The detection limit for TNF-alpha and IL-10 and CRP were 1.7 pg/mL and <1 pg/mL, and 0.2 mg/L respectively (as reported by the manufacturer). All reagents were applied according to the manufacturer's instructions.

### Data processing and statistical analysis

All analyses were performed using PASW Statistics 17.0.2 software (SPSS Inc, Illinois, USA). The differences between the groups were assessed using one-way analysis of variance (with Bonferroni *post hoc *testing). For data that were not distributed normally, the non-parametric Kruskall Wallis and the Mann-Whitney tests were applied. Correlations between continuous parameters were evaluated using the Spearman test. To allow for statistical analysis, values below the detection limit for Hsp70, IL-6 and IL-10 were substituted by one unit below the respective detection limit value (i.e. 0.19 ng/mL for Hsp70, 0.15 pg/mL for high sensitivity IL-6, and 0.99 pg/mL for IL-10). In order to bring together the results for IL-6 obtained via the standard and high-sensitivity assays a method of ranking was applied whereby the values obtained with the high-sensitivity kit were ranked from low to high followed by the values obtained with the standard kit from low to high. Differences between the groups in the proportion of subjects with detectable or undetectable serum levels of Hsp70 and cytokines were analysed using Chi-square. A p-value (2 sided) <0.05 was considered as statistically significant.

## Results

The characteristics of the three study groups at enrolment are given in table 1. The main diagnosis justifying the hospitalisation of the geriatric patients is given in table 2. As expected, the elderly patients and the community-dwelling elderly had significantly higher comorbidity and medication intake than the young adults. The mean age was significantly higher in the patient group compared with the community-dwelling elderly group. On the other hand, the body mass index was significantly higher in the community-dwelling elderly than in the young adult group. Only the elderly patient group showed functional (an increased bADL score) and mental decline (a low MMSE score).

**Table 1:** General characteristics of the participants

**Parameter**	**Geriatric patients**		**Community-dwelling elderly**		**Young adults**	
	
	**Male**	**Female**	**Male**	**Female**	**Male**	**Female**
	**N = 30**	**N = 60**	**N = 49**	**N = 51**	**N = 49**	**N = 51**
**Age **(years)	83.4 ± 5.5*	83.3 ± 5.6*	74.7 ± 4.7^†^	74.2 ± 4.5^†^	23.4 ± 3.3	22.6 ± 2.5
**Height **(metres)	1.71 ± 0.08^†^	1.59 ± 0.05^†^	1.72 ± 0.05^†^	1.60 ± 0.06^†^	1.81 ± 0.08	1.69 ± 0.05
**Weight **(kg)	73.5 ± 11.7	62.2 ± 10.84^$^	78.7 ± 9.9	70.0 ± 12.1^†^	76.7 ± 13.3	61.5 ± 7.7
**BMI **(kg/m^2^)	25.1 ± 3.8	24.6 ± 4.1*	26.7 ± 3.2^†^	27.2 ± 4.3^†^	23.4 ± 3.8	21.7 ± 2.4
**Medication **(number)	9.4 ± 4.7*	8.2 ± 3.8*	2.6 ± 1.8^†^	2.6 ± 1.8^†^	0.3 ± 0.8	0.9 ± 0.9
**Comorbidity **(number)	7.0 ± 2.8*	6.4 ± 2.9*	1.4 ± 1.0^†^	1.4 ± 1.1^†^	0.1 ± 0.4	0.0 ± 0.1
**bADL **(score on 24)	12.1 ± 4.2*	13.2 ± 5.4*	6.0 ± 0.0	6.0 ± 0.0	6.0 ± 0.0	6.0 ± 0.0
**MMSE **(score on 30)	23.1 ± 5.2		n/a	n/a	n/a	n/a

Values expressed as mean ± standard deviation, BMI = body mass index; bADL = basic activities of daily life; MMSE = mini-mental state examination; n/a = not available, significantly different *from community-dwelling elderly and young adults, ^†^from young adults, ^$^from community-dwelling elderly (all One-way ANOVA with Bonferroni *post-hoc *test p < 0.05).

**Table 2:** Main diagnosis and Hsp70 serum concentrations among the elderly patients

Diagnosis	Hsp70	Inflam/Non-inflam
	*Median [IQR]*	*N*
**Infectious disease**	0.42 [0.19; 0.76]	17/1
**Cardiovascular disease**	0.19 [0.19; 0.39]	11/5
**Neuropsychological disorder**	0.19 [0.19; 0.21]	4/7
**Cancer**	0.19 [0.19; 0.55]	6/4
**Musculoskeletal disorder**	0.21 [0.19; 1.54]	6/4
**Gastrointestinal disorder**	0.19 [0.19; 0.19]	5/4
**Cerebrovascular disease**	0.19 [0.19; 0.19]	2/2
**Haematological disorder**	0.19 [0.19; 0.88]	0/3
**Respiratory disorder**	0.35 [0.19; 1.81]	1/2
**Rheumatological disorder**	0.19	1/1
**Diabetes mellitus**	0.19	1/0
**Frailty**	0.24	1/0
**Otorhinolaryngical disease**	0.23	0/1
**Urological disorder**	0.19	1/0

IQR: interquartile range (percentile25; percentile75); no significant difference in Hsp70 concentration between the various diagnostic groups (Kruskall Wallis test p = 0.14; performed for those diagnostic groups with >4 participants); Inflam = inflammatory (CRP serum concentration ≥5 mg/L).

**Figure 1:**
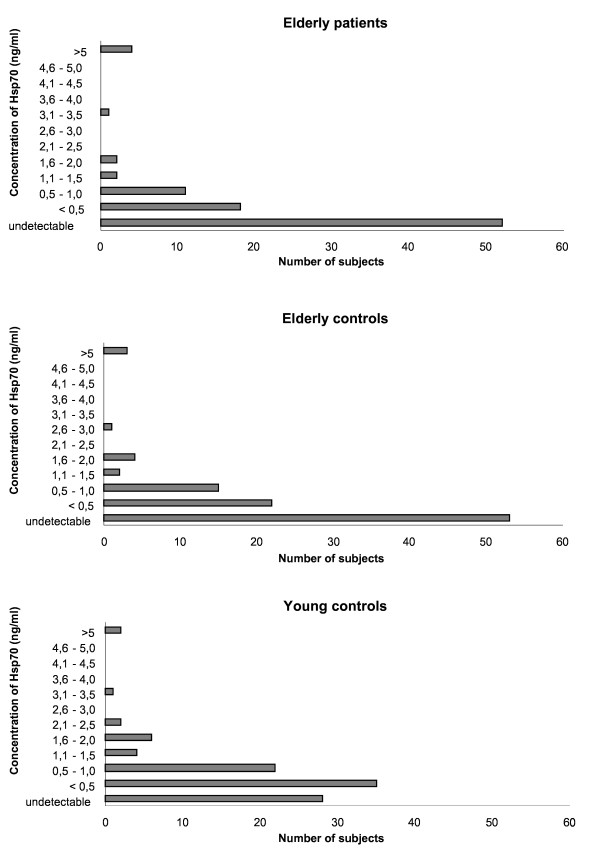
**Hsp70 concentrations in the various categories of participants**. Serum Hsp70 levels were significantly higher in the young compared to the elderly subjects. However, when participants with undetectable Hsp70 serum levels - as well as the few outliers - were excluded, the values in the three groups were not significantly different (Kruskall Wallis: p = 0.114).

Since there was no significant gender difference observed in the levels of Hsp70, IL-6, IL-10, TNF-alpha and CRP, the results from both sexes were pooled. As shown in table 3, the median serum levels of Hsp70 were significantly different for geriatric patients, the community-dwelling elderly and young adults (p < 0.001). This difference was mainly due to the significantly higher number of older participants (more pronounced for the non-inflammatory patients than for the community-dwelling elderly people) for whom the Hsp70 serum values were below the detection limit (p < 0.001). In fact, when participants with undetectable Hsp70 serum levels - as well as the few outliers were not taken into consideration - the values in the three groups were not significantly different (Kruskall Wallis: p = 0.114; see figure 1). Both IL-6 and TNF-alpha serum levels were significantly higher in the community-dwelling elderly compared to the young controls. These cytokines were also significantly higher in the patients compared to the controls. Conversely, the serum levels of IL-10 were significantly lower in the elderly participants compared to the young controls (see table 3).

**Table 3:** Hsp70 and cytokine serum concentrations

Parameter	Infl. Geriatric Patients N = 56	Non-infl. Geriatric Patients N = 34	Community-Dwelling Elderly N = 100	Young Adults N = 100
**Hsp70 (ng/mL)**				
Median [IQR]*	0.22 [0.19; 0.58]	0.19 [0.19; 0.21]^†^	0.19 [0.19; 0.50]^‡^	0.40 [0.19; 0.67]^‡$^
Detectable/Undetectable*	29/27	9/25^†^	47/53^‡^	72/28 ^† ^^‡ ^^$^
**IL-6 (pg/mL Standard)**				
*Median [IQR] *^a^*	27.03 [16.38; 48.84]	12.78 [8.95; 16.88]^†^	13.15 [9.99; 16.99]^†^	9.29 [8.56; 11.97]^†$^
Detectable/Undetectable*	46/10	13/21^†^	28/72^†^	19/81 ^† ^^‡^
**IL-6 (pg/mL Hs)**				
*Median [IQR] *^a^*	3.95 [2.69; 6.82]	4.32 [2.49; 4.91]	2.09 [1.29; 3.27]^†‡^	0.89 [0.70; 1.46]^†‡$^
Detectable/Undetectable*	10/0	21/0	71/1	63/18^‡^
**TNF-alpha (pg/mL)**				
Median [IQR]*	15.65 [11.87; 21.66]	12.93 [10.06; 17.99]^†^	12.49 [10.81; 15.73]^†^	12.07 [10.09; 13.87]^†$^
*Detectable/Undetectable*	56/0	34/0	100/0	100/0
**IL-10 (pg/mL)**				
Median [IQR]*	0.99 [0.99; 2.12]	0.99 [0.99; 0.99]^†^	0.99 [0.99; 1.81]^‡^	1.97 [0.99; 2.97]^†‡$^
Detectable/Undetectable*	19/37	5/29^†^	45/55^‡^	64/36^†‡$^

IQR = interquartile range (percentile25; percentile 75); Infl = inflammatory (CRP serum concentration ≥5 mg/L); ^*a*^Values based on samples with detectable concentrations only; Standard = standard IL-6 kit with a detection limit of 7.8 pg/mL; Hs = high-sensitivity IL-6 kit with a detection limit of 0.16 pg/mL.

*Significant differences among groups (Kruskall-Wallis test or Chi-Square test p < 0.001); significantly different from ^†^Inflammatory geriatric patients, ^‡ ^Non-inflammatory geriatric patients, ^$ ^Community-dwelling elderly (Mann-Whitney U test or Chi-Square test p < 0.05).

We found that in the control subjects age was related negatively with Hsp70 and IL-10, and positively with IL-6 and TNF-alpha serum levels (see table 4). In the elderly patients, a positive association was observed between the Hsp70 serum levels and those of IL-6 (r = 0.287; p < 0.05) and of CRP (r = 0.38; p < 0.001). Among these patients, those without detectable Hsp70 levels gave lower values for IL-6 (p = 0.008) and CRP (respectively median [inter-quartile range] 9.50 [4.11; 22.81] pg/mL and 5.59 [1.85; 23.6] mg/L) than those with detectable levels (respectively 19.02 [8.95; 45.07] pg/mL and 24.85 [5.00; 97.00] mg/L). No such relationships were found for the elderly and young control groups. Positive relationships were noted among the cytokines, IL-6, TNF-alpha and IL-10, in both the patient and control populations.

**Table 4:** Relationship between the inflammatory parameters and Hsp70 in patients and control subjects

	Age (years)	CRP (mg/L)	TNF-alpha (pg/mL)	**IL-6 (Rank order) **^***a***^	IL-10 (pg/mL)
**Hsp70 (ng/mL)**					
Patients (n = 90)	-0.106	0.379**	0.145	0.287**	0.045
Controls (n = 200)	-0.194**	NA	-0.035	-0.118	-0.041
**IL-10 (pg/mL)**					
Patients (n = 90)	-0.051	0.223*	0.327**	0.234*	
Controls (n = 200)	-0.185**	NA	0.162*	0.073	
**IL-6 (Rankorder)**^***a***^					
Patients (n = 90)	0.117	0.699**	0.354**		
Controls (n = 200)	0.421**	NA	0.332**		
**TNF-alpha (pg/mL)**					
Patients (n = 90)	0.110	0.303**			
Controls (n = 200)	0.149*	NA			
**CRP (mg/L)**					
Patients (n = 90)	-0.029				
Controls (n = 200)	NA				

NA = not applicable; ^*a*^Serum samples with the concentration of IL-6 lower than 7.8 pg/mL, which was the lower detection limit for the standard IL-6 kit, were re-analysed using the high-sensitive IL-6 kit with a detection limit of 0.16 pg/mL. In order to bring results for IL-6 obtained by the standard and high-sensitivity assays together, a ranking method was applied whereby the values obtained with the high-sensitivity kit were ranked from low to high followed by the values obtained with the standard kit from low to high. Values represent Spearman's rho correlation coefficients, * p < 0.05, ** p < 0.001.

We found no significant differences in the levels of serum Hsp70 among the various diagnoses for the elderly patients (see table 2). However, since patients with infectious disease tended to have higher Hsp70 values, patients were further categorised according to their inflammatory profile. When inflammation was present (as assessed by CRP serum levels ≥5 mg/L), significantly (p = 0.007) higher Hsp70 values were observed (see table 3). Among the elderly patients there was a negative correlation (r = -0.37; p = 0.004; N = 55) between the MMSE score and the Hsp70 serum values. As regards the bADL score, a positive correlation (r = 0.25; p = 0.017; N = 89) was found with the Hsp70 serum values.

## Discussion

In this study, we provide new evidence regarding the age-related differences in circulating Hsp70 levels by comparing 190 elderly persons - stratified for clinical profile - with 100 healthy young controls. Our results show an age-related decrease in the serum levels of Hsp70. The main difference was in the proportion of participants for whom the Hsp serum levels were below the detection limit, which was significantly higher in the elderly controls and patients when compared to their young counterparts (both p < 0.001). When only the subjects with detectable Hsp70 levels were taken into consideration and the few outlying high values were discarded, there was no significant difference between the levels of Hsp70 for the young controls compared to the ambulatory elderly. Thus, contrary to our observations for intracellular Hsp70 [16], it can be stated that circulating Hsp70 decreases with aging in normal older subjects. The results of a previous smaller-scale study [4] concord with the age-related decrease in serum Hsp70 we report here. Other studies dealing with the effect of age on circulating Hsp70 either failed to include subjects over 50 years of age [25], or focused on exceptional longevity in centenarians [26]. Although the mechanisms underlying the age-related effects on circulating Hsp70 are largely unknown, evidence from *in vitro *studies suggest that age-related differences in the production of intracellular Hsp70 might reflect the limited transcription of the heat shock genes, which has been confirmed as due to reduced activation and binding of the HSF 1 to the HSE and/or an impairment in the post-translational processing of Hsp70 mRNA, resulting in its impaired nuclear transport [4].

In geriatric non-inflammatory patients, Hsp70 was less detectable and circulating levels were lower than for the healthier community-dwelling older subjects. However, this was not the case for inflammatory geriatric patients who had higher circulating Hsp70 values than the non-inflammatory ones. A positive association was observed between the Hsp70 serum levels and the levels of CRP and IL-6 in patients, in agreement with previous observations we made about geriatric patients [19,20]. *In vitro *studies [27,28] have shown that IL-6 can up-regulate the production of Hsp through signal transduction by the transcription factors Nuclear Factor IL-6 and the Signal Transducer and Activator of Transcription (STAT)-3. Similarly, other cytokines, including TNF-alpha [29,30] and INF-gamma through STAT-1 signalling [31], have been shown to stimulate the production of Hsp70. In the present study, neither TNF-alpha nor IL-10 serum levels indicated a relationship with circulating Hsp70.

As expected, the serum levels of both TNF-alpha and IL-6 increased significantly with age in the combined control groups, while the IL-10 serum level decreased. An age-related decrease in IL-10 was reported in mice by Ye and Johnson [32]. Many other studies have also described increased TNF-alpha and IL-6 concentrations with age [15,33,34], which were associated with a spectrum of age-related conditions, including low-grade inflammation, frailty and functional decline [35]. The fact that IL-6 was related to Hsp70 only in the patients and not in the control participants might be explained by differences between patients and controls in the complex cytokine environment.

In the present study, a significant difference was noted in Hsp70 levels between elderly controls and non-inflammatory elderly patients. It is difficult to draw any parallels between the mechanisms leading to Hsp70 production in patients and those involved in the more restrained changes associated with aging in normal individuals. In patients, and depending on the pathological situation, differential associations between Hsp70 and disease status have been reported. For instance, higher circulating levels of Hsp70 have been shown in patients with chronic heart failure [36] and coronary artery diseases [37], which was suggested to be associated with stretch and decreased myocyte shortening [38]. Conversely, reduced Hsp70 levels were observed in patients with chronic obstructive pulmonary disease [39] and in AIDS patients [40]. More notably, in the present study there was a broad case mix, which might have neutralised specific associations between Hsp70 and a particular pathology.

It has been reported that in the absence of serious inflammatory conditions, low levels of serum Hsp70 are associated with successful biological aging [26] and might reflect a strong anti-inflammatory status of an individual's immune system [41]. In the elderly patient group, Hsp70 was associated positively with clinical markers of frailty. Indeed, Hsp70 serum levels were significantly higher in the most frail patients, as documented by the positive correlation with the level of physical dependency (bADL score: r = 0.24; p = 0.022) and the negative correlation with cognitive functioning (MMSE score: r = -0.37; p = 0.004). These results are in accordance with our previous report on elderly nursing-home residents, showing higher circulating Hsp70 with increasing dependency [42]. Also, in the study on elderly persons with exceptional longevity, mentioned above [26], it appeared that higher circulating Hsp70 levels indicated a poorer clinical condition. To our knowledge, relationships between dementia and circulating Hsp70 have not been established; only one report mentions increased plasma Hsp70 levels in patients with vascular mild cognitive impairment [43]. The exact mechanisms behind such a relationship remain uncertain, but might involve increased oxidative stress [44-46]. Following these observations, it can be hypothesised that Hsp70 in serum has beneficial effects during acute elevations (of short duration), but is related to negative clinical conditions when chronically elevated.

## Conclusions

In conclusion, the present study shows that the serum concentration of Hsp70 decreases with age in a normal population, adding further evidence to the concept that the capacity to produce Hsp70 decreases with aging. In addition, our study suggests that increased serum levels of Hsp70 are associated with frail health, as documented by worse bADL and MMSE scores in patients. Inflammatory pathology in elderly patients is accompanied by higher Hsp70 levels. These results pave the way for a more detailed exploration of Hsp70 in frailty. In particular, understanding whether Hsp70 plays a role in frailty or is only a consequence of frailty, is intriguing.

## Authors' contributions

IB and TM conceived and designed the study. RN performed the assays, analysed the data, and wrote the manuscript. TM participated substantially in the analysis and writing of the manuscript. IB coordinated the recruitment of participants, data collection and analysis. OO participated in the determination of cytokines. KVP participated in the diagnosis and recruitment of patients. CD made significant contributions regarding the technical aspects of the manuscript. All authors read and approved the final manuscript.
